# Antitumor effect of plant-produced anti-CTLA-4 monoclonal antibody in a murine model of colon cancer

**DOI:** 10.3389/fpls.2023.1149455

**Published:** 2023-08-29

**Authors:** Christine Joy I. Bulaon, Narach Khorattanakulchai, Kaewta Rattanapisit, Hongyan Sun, Nuttapat Pisuttinusart, Richard Strasser, Shiho Tanaka, Patrick Soon-Shiong, Waranyoo Phoolcharoen

**Affiliations:** ^1^ Center of Excellence in Plant-Produced Pharmaceuticals, Chulalongkorn University, Bangkok, Thailand; ^2^ Department of Pharmacognosy and Pharmaceutical Botany, Faculty of Pharmaceutical Sciences, Chulalongkorn University, Bangkok, Thailand; ^3^ Graduate Program of Pharmaceutical Sciences and Technology, Faculty of Pharmaceutical Sciences, Chulalongkorn University, Bangkok, Thailand; ^4^ Baiya Phytopharm Co., Ltd, Bangkok, Thailand; ^5^ GemPharmatech Co., Ltd, Nanjing, China; ^6^ Department of Applied Genetics and Cell Biology, University of Natural Resources and Life Sciences, Vienna, Austria; ^7^ ImmunityBio, Inc., Culver City, CA, United States

**Keywords:** cytotoxic T lymphocyte-associated protein 4, 2C8, anti-CTLA-4 antibody, *Nicotiana benthamiana*, cancer immunotherapy

## Abstract

Cytotoxic T lymphocyte-associated protein 4 (CTLA-4) is an immune checkpoint regulator exclusively expressed on T cells that obstructs the cell’s effector functions. Ipilimumab (Yervoy®), a CTLA-4 blocking antibody, emerged as a notable breakthrough in modern cancer treatment, showing upfront clinical benefits in multiple carcinomas. However, the exhilarating cost of checkpoint blockade therapy is discouraging and even utmost prominent in developing countries. Thereby, affordability of cancer care has become a point of emphasis in drug development pipelines. Plant expression system blossomed as a cutting-edge platform for rapid, facile to scale-up, and economical production of recombinant therapeutics. Here, we describe the production of an anti-CTLA-4 2C8 antibody in *Nicotiana benthamiana*. ELISA and bio-layer interferometry were used to analyze antigen binding and binding kinetics. Anticancer responses in vivo were evaluated using knocked-in mice implanted with syngeneic colon tumor. At 4 days post-infiltration, the antibody was transiently expressed in plants with yields of up to 39.65 ± 8.42 μg/g fresh weight. Plant-produced 2C8 binds to both human and murine CTLA-4, and the plant-produced IgG1 also binds to human FcγRIIIa (V158). In addition, the plant-produced 2C8 monoclonal antibody is as effective as Yervoy® in inhibiting tumor growth in vivo. In conclusion, our study underlines the applicability of plant platform to produce functional therapeutic antibodies with promising potential in cancer immunotherapy.

## Introduction

Cancer is a multifaceted disease that exerts a heavy toll on society. It is still the leading cause of fatality worldwide with the developing nations shouldering most of the burden. Since then, cancer management with surgery, radiotherapy and chemotherapy has provided a chance of cure and achieved satisfactory results (Arruebo et al., 2011). However, shortcomings of these current cancer modalities including post-operative recurrence, resistance, and side effects limit their clinical success. In attempt to augment the traditional methods for cancer therapy, developments in the treatment landscape represent major areas of focus. The worthwhile advancement in cancer therapeutics have shifted the paradigm for cancer treatment. Among these, cancer immunotherapy has drawn significant attention in the oncology field. The concept of immunotherapy involves variety of approaches aimed at harnessing the immune system to target cancerous cells. The efficacy of these immune-based therapies is being actively evaluated in several clinical trials (Behrouzieh et al., 2021; Juat et al., 2022; Liu et al., 2022) and has shown considerable promise in a number of human malignancies, for which immunotherapy is regarded as a standard of care.

Immune checkpoint blockade (ICB) therapy has become one of the most common options for cancer immunotherapy within the therapeutic armamentarium. Several immune checkpoint proteins, such as programmed cell death protein 1 (PD-1), programmed cell death ligand 1 (PD-L1), and cytotoxic T lymphocyte-associated protein 4 (CTLA-4), enable peripheral immune tolerance under physiological conditions although often become coopted in the cancer context. Immune checkpoint inhibitors (ICIs) could reverse T cell enervation and restore antitumor effector functions, ultimately leading to cancer eradication. ICB therapy has gained prominence and is being approved in multiple cancer indications largely due to durable response (Tang et al., 2018) and improved safety (Khan et al., 2018) observed in clinical practice. The CTLA-4 checkpoint receptor provided the first target for antibody-based immunotherapy and is a homolog of CD28 receptor, which both belong to the immunoglobulin superfamily on T cells. It is postulated that CTLA-4 can negatively regulate T cell signaling by directly antagonizing CD28. Due to the higher affinity binding of CTLA-4 to CD80 and CD86, CTLA-4 can outcompete CD28 for co-stimulatory ligands, thus inhibiting T cell activation. Additionally, CTLA-4 is constitutively expressed on regulatory T cells (Tregs), indicating that CTLA-4 receptor plays a vital role for direct and indirect immunosuppressive activity of Tregs. To that end, the relevance of CTLA-4 function led to the development of inhibitory antibodies obturating its actions to enhance T cell antitumor responses. The two approved monoclonal antibodies (mAbs) that target CTLA-4 are Ipilimumab (Yervoy^®^, MDX-010, BMS-734016) and Tremelimumab (tremelimumab-actl; IMJUDO^®^). They are currently used as monotherapy or in combination to treat a variety of malignant tumors, including hepatocellular carcinoma, non-small cell lung cancer, melanoma and other solid tumors (Cameron et al., 2011; Cabel et al., 2017; Morse et al., 2019; Keam, 2023). With the substantial number of preclinical and clinical studies, anti-CTLA-4 mAb has proven the unprecedented potentials of ICB immunotherapy. Interestingly, while cancer remains a universal concern, the staggering high production cost of recombinant blocking antibodies is expected to impact in low- and middle-income countries.

The use of plant expression systems to produce pharmaceutical and non-pharmaceuticals is constantly expanding and gaining recognition in the research (Bulaon et al., 2020; Shanmugaraj et al., 2020a; Rattanapisit et al., 2021; Shanmugaraj et al., 2021) and manufacturing industries (Nosaki et al., 2021). Some of its key advantages include rapid and ease of cultivation, scalability, lack or low risk of pathogen contamination, ability to carry out posttranslational modifications and cost-effectiveness (Nandi et al., 2016; Shanmugaraj et al., 2020a; Nosaki et al., 2021). For several years, mammalian cell cultures (i.e., CHO cells) have been the dominant expression system for mAbs. Nonetheless, the plant-based facility is projected to lower operating expenses and cost of goods sold compared to the CHO facility (Kelley, 2009; Petrides et al., 2014; Nandi et al., 2016). The downstream processing costs for a mAb produced using a conventional CHO cell platform at a high production capacity (1,000 kg/year) were estimated to be $232/g. Even at a much lower production capacity (600 kg/year), the plant-derived platform has a cost of goods sold of ∼$99/g, which includes both upstream and downstream process operations, corresponding to decrease of over 50% in manufacturing costs. According to the presented techno-economic model (Nandi et al., 2016), plant-based expression technology is a promising platform for the production of mAbs. Our team (Rattanapisit et al., 2019; Phakham et al., 2021; Yiemchavee et al., 2021; Phetphoung et al., 2022) have previously expressed recombinant immunotherapeutic antigens and antibodies in plants, providing proof of principle for its viability. To attenuate striking disparities and improve access to treatment in impoverished countries, plant-based approach offers a competitive platform for the development of immunomodulatory agents for cancer immunotherapy. Herein, we describe the rapid transient expression of an anti-CTLA-4 antibody, 2C8, in *Nicotiana benthamiana.* This plant-produced antibody was purified and characterized for its physicochemical and functional properties. We found that the plant-produced anti-CTLA-4 2C8 mAb binds to human and murine CTLA-4 proteins as well as to one of the Fcγ receptors (FcγRs). Further, this plant-produced anti-CTLA-4 2C8 mAb demonstrated similar antitumor efficacy with the commercial anti-CTLA-4 mAb (Yervoy^®^) in a humanized mouse tumor model. These results imply that the plant-produced anti-CTLA-4 mAb may have comparable therapeutic potentials to clinically effective Ipilimumab.

## Materials and methods

### Expression vector constructs

The nucleotide sequences encoding the variable regions of anti-CTLA-4 2C8 heavy chain (HC) and light chain (LC) (Wang et al., 2017) were codon optimized *in silico* for expression in *N. benthamiana* and synthesized commercially (Bioneer, South Korea). To construct the full-length HC and LC of 2C8 mAb, the plant-optimized variable HC and LC genes were fused with the constant regions of human immunoglobulin 1 (IgG1) gamma chain and kappa chain, respectively. In addition, both the HC and LC of 2C8 mAb harbor a signal peptide at the N-terminus and a SEKDEL retention peptide at the C-terminus of HC. The HC and LC of anti-CTLA-4 2C8 mAb were subcloned using *Xba*I and *Sac*I restriction enzymes in a plant expression vector (pBYR2e) (Chen et al., 2011; Diamos and Mason, 2019). The constructed anti-CTLA-4 pBYR2e-2C8 HC and pBYR2e-2C8 LC expression vectors were introduced into *Agrobacterium tumefaciens* strain GV3101 by electroporation and the recombinant colonies were selected on appropriate antibiotic (50 mg l^-1^ kanamycin; 50 mg l^-1^ gentamycin; 50 mg l^-1^ rifampicin) supplemented Luria Bertani (LB) agar plate. All expression vectors (pBYR2e-2C8 HC and pBYR2e-2C8 LC) in *A. tumefaciens* cells were sequenced verified (U2Bio, Thailand) prior to infiltration in *Nicotiana benthamiana* plants.

### Transient expression in *Nicotiana benthamiana*

The *Agrobacterium* harboring either the pBYR2e-2C8 HC or pBYR2e-2C8 LC plasmids were cultivated on liquid LB media containing 50 mg l^-1^ kanamycin, 50 mg l^-1^ gentamycin, and 50 mg l^-1^ rifampicin overnight with shaking (200 rpm) at 28°C in the dark. The *Agrobacterium* cells were pelleted and resuspended with 1X infiltration buffer (10 mM 2-N-morpholino-ethanesulfonic acid (MES); 10 mM MgSO_4_) at pH 5.5. A 1:1 ratio of HC and LC cultures were mixed to get a final OD_600_ of 0.2.

Approximately 6–8-week-old *N. benthamiana* plants were grown in a plant room under controlled conditions (16 h light and 8 h dark cycle at 28°C) and used for expression. The method for small-scale infiltration in plants was adopted from (Phakham et al., 2021). Briefly, the HC and LC genes were co-agroinfiltrated into *N. benthamiana* leaves using needle-less syringes. The infiltrated plant leaves were maintained and harvested within 2-, 3-, 4-, 5-, and 6-days post-infiltration (dpi) to initially evaluate the expression level of plant-produced anti-CTLA-4 antibody.

### Isolation and quantification of plant-produced anti-CTLA-4

Infiltrated leaf samples collected at different dpi were chopped into small leaf discs and pooled to obtain ~20-50 mg leaf fresh weight. The leaves were extracted by a tissue lyser (Retsch, Model: MM 400) with stainless steel grinding balls (diameter 3 mm) (Retsch, Germany) in 200 µl 1X phosphate-buffered saline (PBS: 137 mM NaCl; 2.7 mM KCl; 4.3 mM Na_2_HPO_4_; 1.47 mM KH_2_PO_4_) at pH 7.4. The resultant crude extract was subjected to centrifugation (20,000 × *g* for 5 min) at 4°C and the supernatant was analyzed to determine the yield of plant-produced 2C8 mAb by enzyme-linked immunosorbent assay (ELISA).

Briefly, a 96-well microtiter plate (3690, Corning, United States) was coated with 25 µl of goat anti-human IgG, Fc specific antibody (dilution 1:1000) (ab97221, Abcam, United Kingdom) and incubated overnight at 4°C. The ELISA plate was washed with 1X PBST (PBS with 0.05% (v/v) Tween 20) for three times and blocked with 5% (w/v) skim milk in 1X PBS for 2 h at 37°C. After washing, two-fold serial dilutions of standard human IgG1 isotype control (ab206198, Abcam, United Kingdom) and plant crude extracts were added and incubated for 2 h at 37°C. Then, the plate was washed and treated with peroxidase-conjugated goat anti-human kappa antibody (dilution 1:2500) (2060-05, SouthernBiotech, United States) for 1 h at 37°C. After three-time washes with 1X PBST, the plate was developed using TMB one solution substrate (Promega, United States) and the reaction was stopped with 1M H_2_SO_4_. The absorbance was measured at 450 nm by a microplate reader (Hercuvan, Model: NS-100).

### Purification of plant-produced anti-CTLA-4

The production of plant-produced anti-CTLA-4 2C8 mAb was scaled up by vacuum infiltration. In brief, *Agrobacterium* cultures harboring recombinant HC and LC vectors were mixed together at a ratio of 1:1 in 1X infiltration buffer to achieve an OD_600_ of 0.2. Plant leaves were then submerged in an infiltration medium containing an *Agrobacterium* suspension and infiltrated under vacuum at 600–760 mmHg for 1-2 min. Infiltrated tobacco plants were incubated in an indoor plant room and harvested at optimal dpi. About 70 g of infiltrated *N. benthamiana* leaves were harvested at 4 dpi and homogenized by blender with 140 mL 1X PBS. The plant crude extract was centrifuged (13,000 rpm for 30 min) at 4°C and filtered by a cheesecloth and a 0.22-μm S-Pak filter (Merck Millipore, United States). The clarified supernatant was loaded onto a polypropylene column (diameter 15 mm) (Qiagen, Germany) packed with MabSelect SuRe™ LX protein A resin (Cytiva, Sweden). The column was washed with 1X PBS, and the plant-produced antibody was eluted using a 0.15 M citrate buffer at pH 2.7 and immediately neutralized with 1.5 M Tris-HCl pH 8.8 until a final pH of approximately 7.4. The purified antibody was buffer-exchanged with 1X PBS using a Snakeskin™ dialysis tubing (3.5K MWCO) (Thermo Scientific, United States) and concentrated in Amicon^®^ Ultra (50K) centrifugal filter (Merck, Germany). The purified plant-produced anti-CTLA-4 antibody was quantified by ELISA and characterized in succeeding experiments.

### Sodium dodecyl sulfate polyacrylamide gel electrophoresis and immunoblot analysis

Plant crude extracts and purified antibody samples were separated by SDS-PAGE and analyzed by western blot to confirm the antibody expression. For non-reducing condition, protein samples were mixed with 10X loading buffer (125 mM Tris-HCl at pH 6.8; 12% (w/v) SDS; 10% (v/v) glycerol; 0.001% (w/v) bromophenol blue). Whereas, for reducing condition, protein samples were mixed with 10X loading buffer containing 22% (v/v) β-mercaptoethanol. The proteins were heated for 5 min at 95°C and separated on a 4-15% gel. For visualization of purified plant-produced antibody (~2 µg) on SDS gel, the bands were stained with InstantBlue^®^ dye (Abcam, United Kingdom).

For western blotting, about 20-40 µg total soluble protein of plant crude extracts and 0.1-1.0 µg of purified antibody were transferred to a nitrocellulose membrane (Bio-Rad, United States). Then, the membrane was blocked with 5% (w/v) skim milk in 1X PBS. The blot membranes were incubated either with peroxidase conjugated goat anti-human IgG gamma chain antibody (dilution 1:5000) (2040-05, SouthernBiotech, United States) or peroxidase-conjugated goat anti-human kappa antibody (dilution 1:5000) (2060-05, SouthernBiotech, United States) for the detection of HC and LC of plant-produced anti-CTLA-4 2C8 mAb. A non-infiltrated plant crude extract was used as a negative control. The membranes were washed with 1X PBST and exposed to a medical X-ray green film (Carestream, United States) using enhanced chemiluminescence substrate solution (Promega, United States).

### Size exclusion chromatography

To determine the antibody purity and aggregation status of the antibody, the plant-produced anti-CTLA-4 mAb was injected into a SEC BEH 200 column (4.6 x 300 mm, 2.5 μM particle size) (Waters, United States) attached on an UHPLC system (Waters, United States). The 1X PBS buffer at pH 7.4 was used as an elution buffer and operated at 0.3 mL/min flow rate. The column was maintained at 25°C and the sample was run for 20 min. The UV absorbance signal was monitored at 280 nm and the peak was integrated by using Empower 3 software (Waters, United States).

### *N*-Glycan analysis

Liquid chromatography-electrospray ionization-mass spectrometry (LC-ESI-MS) was performed to assess the *N*-glycan profile of our purified plant-produced anti-CTLA-4. In brief, the antibody was initially subjected to reducing SDS-PAGE and then the protein band of heavy chain was cut into slices, S-alkylated and proteolytically digested. The tryptic glycopeptides were analyzed by LC-ESI-MS following the protocol of (Strasser et al., 2008).

### CTLA-4 binding ELISA

Recombinant human CTLA-4 protein (huCTLA-4) (11159-H08H, SinoBiological, China) and mouse CTLA-4 protein (muCTLA-4) (50503-M08H, SinoBiological, China) were used to verify the binding activity of plant-produced anti-CTLA-4 2C8 mAb to its target. Briefly, microtiter plates (3690, Corning, United States) were coated at a concentration of 2 µg/mL overnight at 4°C. Then, the plates were washed with 1X PBST and blocked with 5% (w/v) skim milk in 1X PBS for 1 h at 37°C. Two-fold serial dilutions starting from 8 µg/mL of plant-produced anti-CTLA-4 2C8 antibody or plant-produced irrelevant antibody (anti-PD-1) (Rattanapisit et al., 2019) or human IgG1 isotype control (ab206198, Abcam, United Kingdom) were added into the plates and incubated for 2 h at 37°C. After three-time washes with 1X PBST, the plates were incubated with peroxidase-conjugated goat anti-human kappa antibody (dilution 1:2500) (2060-05, SouthernBiotech, United States) for 1 h at 37°C. The plates were washed and then developed using TMB one solution substrate (Promega, United States) for 15 min at room temperature, before quenching with 1 M H_2_SO_4_. The absorbance was measured at 450 nm by a microplate reader (Hercuvan, Model: NS-100).

### Equilibrium dissociation constant (*K*_D_) determination by bio-layer interferometry

BLI buffer used in all experiments was 10 mM HEPES, 150 mM NaCl, pH 7.4, with 0.02% Tween 20, and 0.1% BSA. Commercially purchased recombinant human and mouse CTLA-4 proteins (SinoBiological, China) and human FcγRIIIa (V158) protein (R&D systems, United States) were used in the experiments. For determining the binding affinity for huCTLA-4 and muCTLA-4, plant-produced anti-CTLA-4 2C8 mAb was immobilized on anti-hIgG Fc Capture (AHC) biosensors (Sartorius Corporation, Germany). A concentration series of 100, 50, 25, 12.5 nM of huCTLA-4 and a concentration series of 100, 25, 6.25, 3.13 of muCTLA-4 were used to determine the kinetic constants (*k*_on_, *k*_off_, *K*_D_) for huCTLA-4 and muCTLA-4, respectively, using 1:1 binding curve fit. For determining binding affinity for huFcγRIIIa (V 158), the receptor protein was loaded on the HIS1K sensor, and a concentration series of 4000, 2000, 1000, 500, 250, 125, 62.5 nM of anti-CTLA-4 2C8 was used to determine *K*_D_ using steady state analysis.

### *In vivo* analysis in mouse tumor model

The study protocol (Animal Protocol No. GPTAP20220128-4) was reviewed and approved by the Institutional Animal Care and Use Committee (IACUC) of GemPharmatech Co., Ltd., China prior execution. The procedures involving care and utilization of animals were conducted in accordance with the laws and regulations stated in the Animal Welfare Act and the Association for Assessment and Accreditation of Laboratory Animal Care (AAALAC).

Six to eight weeks old female knocked-in mice were used in this study. Accordingly, humanized mice model (BALB/c-hPD-1/hPD-L1/hCTLA-4) was developed by GemPharmatech, Co., Ltd with experimental animal using license (SYXK (SU) 2018-0027) and experimental animal production license (SCXK (SU) 2018-0008). CRISPR/Cas9 technology was used to replace the extracellular regions of murine PD-1, PD-L1, and CTLA-4 with human PD-1, PD-L1, and CTLA-4 on a BALB/c background. Following, the expression of human proteins was detected in this strain, with no apparent difference in T/B/NK cell proportion observed between wild type and humanized mice. Thereby, this strain can be used to test immune-oncology drugs. BALB/c-hPD-1/hPD-L1/hCTLA-4 were implanted with CT26 murine colon cancer cells expressing humanized PD-L1 (CT26-hPDL1) subcutaneously on the right flank (1×10^6^/100 µL cells). When the average tumor size reached 100.07 mm^3^, all mice (*n* = 18) were randomly allocated into 3 treatment groups (*n* = 6 per group). 1X PBS as vehicle or anti-CTLA-4 antibodies (3 mg/kg), such as plant-produced 2C8 and commercially available Ipilimumab (Yervoy^®^), were administered via., intraperitoneal injection on days 0, 3, 6, 9, 12, 15. To evaluate the treatment efficacy, tumor growth inhibition (TGI) models based on tumor volume (TV) and tumor weight (TW) measurements were utilized. Furthermore, body weight changes were measured by an electronic weighing scale three times in a week.

### Statistical analysis

All data were expressed as mean ± standard deviation (mean ± SD). One way ANOVA followed by multiple comparison procedures was performed. Data were analyzed with SPSS and all tests were two-sided. A *p* value < 0.001 was considered to be statistically significant and noted with ***.

## Results

### Transient expression of 2C8, an anti-CTLA-4 mAb

**Figure 1:**
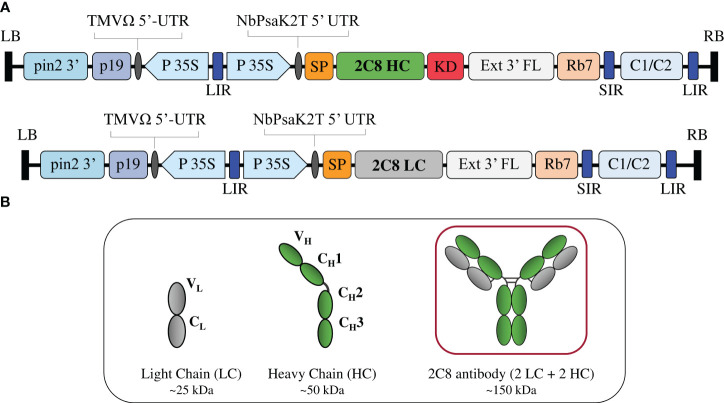
Construction of anti-CTLA-4 2C8 mAb. **(A)** Schematic representation of the gene expression vectors used in the study. The T-DNA elements flanked between the LB (left border) and RB (right border) of pBYR2e comprises of P19: silencing suppressor gene derived from TBSV; TMV 5′ UTR: tobacco mosaic virus Ω 5′ UTR; P35S: CaMV 35S promoter; NbPsaK2T 5′UTR: *Nicotiana* photosystem I reaction center subunit *psaK* 5′UTR; Ext3′FL: 3′ region of the tobacco extension gene; Rb 7: tobacco RB7 promoter; and C1/C2: BeYDV ORFs C1 and C2 encoding for Rep and RepA. The 2C8 HC and 2C8 LC contained DNA sequences encoding for the HC (heavy chain) and LC (light chain) of anti-CTLA-4 2C8 antibody. These expression cassettes are fused either with SP (signal peptide) and/or KD (SEKDEL tag) and inserted between the LIR and SIR (long and short intergenic regions of the BeYDV genome). **(B)** Schematic and structure diagram of HC, LC, and assembled plant-produced anti-CTLA-4 2C8 mAb.

**Figure 2:**
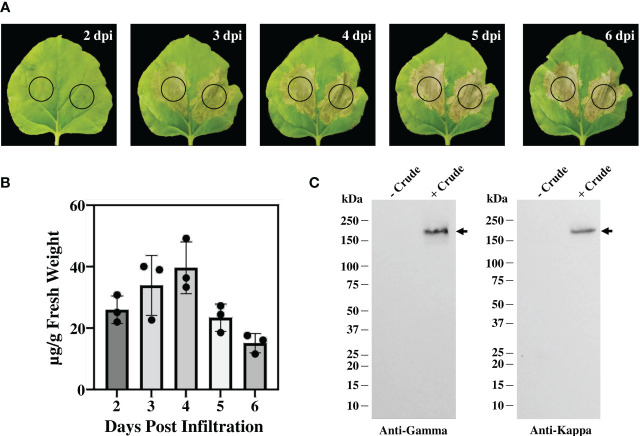
Transient expression of anti-CTLA-4 2C8 mAb in tobacco plants. **(A)** Physical morphology of *N. benthamiana* leaves for 2-6 days after infiltration with *Agrobacterium* carrying pBYR2e-2C8 HC + LC expression vectors. Black circle indicates infiltration site. **(B)** The expression levels of mAb (µg/g fresh weight) in *N. bethamiana* on days 2, 3, 4, 5, and 6 post-infiltration were quantified by ELISA. The data are presented as mean ± SD of three independent experiments. **(C)** The expression of mAb from crude leaf extracts was verified by western blot and detected by either peroxidase-conjugated anti-human IgG gamma chain or anti-human kappa antibody. Numbers on the left indicate molecular weight (kDa). – Crude: non-infiltrated crude extract as the control and + Crude: infiltrated crude extract. Black arrows indicate antibody bands.

The genetic cassettes of anti-CTLA-4 pBYR2e-2C8 HC and pBYR2e-2C8 LC (**Figure 1A**) were co-delivered into *N. benthamiana* by *Agrobacterium-*mediated transformation to produce complete recombinant antibody with 2 HC and 2 LC (**Figure 1B**). Expression of anti-CTLA-4 2C8 mAb was determined over a 5-day timepoints and infiltrated leaves were collected daily from 2 dpi. The morphological changes in *N. benthamiana* leaves at the infiltrated site were examined (**Figure 2A**, indicated in circles). At 3 dpi, visible necrotic lesion was observed, while the surrounding non-infiltrated leaf area showed no necrosis. Necrosis with associated wilting was later developed from 4 to 6 dpi. The effect of dpi on antibody expression level was quantified by ELISA. Infiltrated leaves with evidence of infection or necrosis were collected and used to extract recombinant antibody. Based on the result, the highest expression of plant-produced antibody was achieved at 4 dpi, amounting up to 39.65 ± 8.42 µg/g fresh weight (**Figure 2B**; **Supplementary Table 1**). The presence of plant-produced antibody in crude leaf extracts was confirmed by immunoblotting. Under a non-reduced condition, the infiltrated crude extract (+ Crude) showed a single band at ~150 kDa while non-infiltrated crude extract (- Crude) did not show any detectable band (**Figure 2C**). The HC and LC bands at ~150 kDa could correspond to the expression of a fully assembled antibody when detected by anti-human IgG gamma and anti-human kappa antibodies. Overall, our findings indicated that plant-produced anti-CTLA-4 2C8 mAb was successfully and rapidly produced in plants via., transient expression approach.

### Characterization of anti-CTLA-4 2C8 mAb produced in *N. benthamiana*

**Figure 3:**
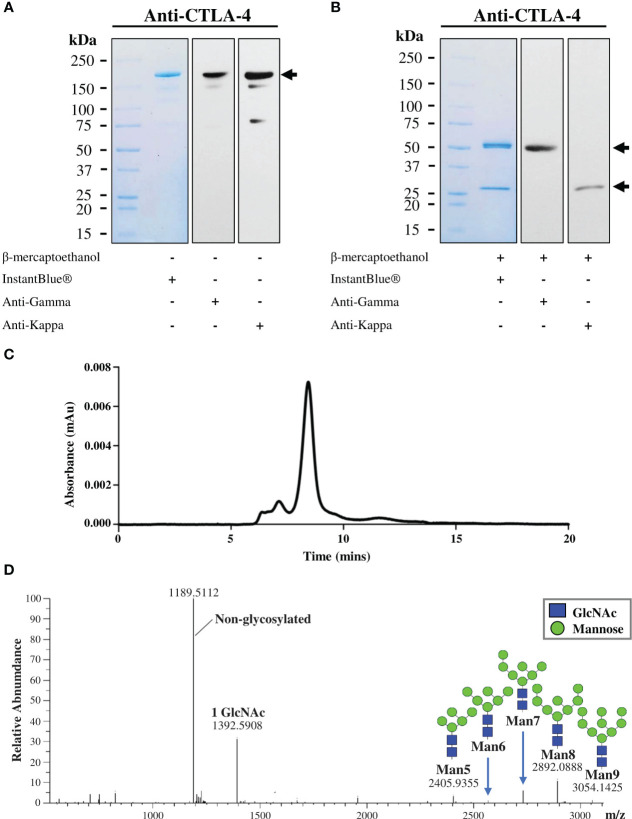
Characterization of purified plant-produced anti-CTLA-4 2C8 mAb. The purified plant-produced antibody was characterized by SDS-PAGE under **(A)** non-reducing and **(B)** reducing conditions. Separated proteins were either visualized by InstantBlue^®^ staining (left) or transferred onto nitrocellulose membrane probed either with peroxidase-conjugated anti-human IgG gamma chain (middle) or anti-human kappa antibody (right). Numbers on the left indicate molecular weight (kDa). –: without β-mercaptoethanol and +: with β-mercaptoethanol. **(C)** Elution profile of purified plant-produced anti-CTLA-4 by size exclusion chromatography. **(D)** The *N*-glycosylation profile of plant-produced glycopeptide EEQYNSTYR (glycosylation site is underlined) from the Fc domain is shown, and the major glycosylated peaks are described. Black arrows indicate antibody bands.

The plant-produced mAb was purified from crude leaf extracts using protein A affinity chromatography and the purity was determined by InstantBlue^®^ staining. The purified plant-produced antibody was further confirmed by immunblot assay using peroxidase-conjugated HC-specific and LC-specific antibodies. Under non-reducing condition, the plant-produced antibody was shown as a single distinct band at ~150 kDa (**Figure 3A**), with trace levels of antibody fragments also observed. The results indicated the assembly of a full length IgG containing two identical HCs and two identical LCs as identified by anti-human gamma and anti-human kappa antibodies. Meanwhile, under reducing condition, protein bands at ~50 kDa and ~25 kDa were detected corresponding to the expected HC and LC of plant-produced antibody (**Figure 3B**). SDS-PAGE and immunoblot results verified the presence of unassembled free HC and LC in the blots containing reducing agent β-mercaptoethanol. To identify intact antibody and aggregates, plant-produced anti-CTLA-4 2C8 mAb was analyzed by SEC separation. **Figure 3C** shows a chromatogram of SEC fractions containing a plant-produced anti-CTLA-4 IgG monomer (major peak) and some observed amounts of aggregated forms (minor peaks). According to our results, plant-produced monoclonal antibody largely assembled and remained as a monomeric species. Moreover, to determine the *N*-glycosylation pattern, plant-produced anti-CTLA-4 2C8 mAb was first digested with trypsin followed by LC-ESI-MS analysis. The attachment of SEKDEL or KD tag to the C-terminus of heavy chain revealed the presence of oligomannosidic *N*-glycans Man_5-9_GlcNAc_2_ on the plant-derived antibody (**Figure 3D**), which is expected for ER-retained proteins.

### Plant-produced anti-CTLA-4 2C8 mAb binds to CTLA-4 and FcγRIIIa (V158)

**Figure 4:**
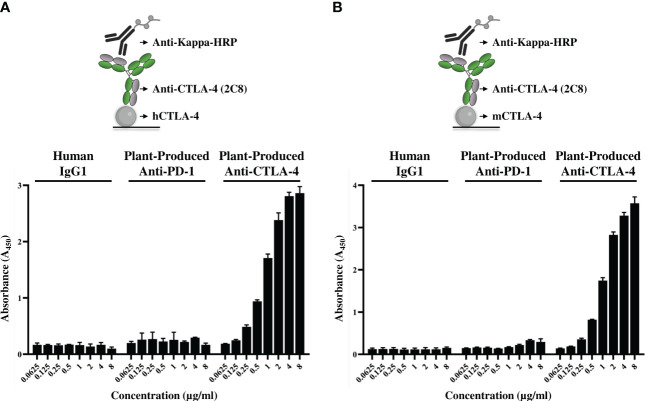
Binding properties of plant-produced anti-CTLA-4 2C8 mAb. The purified plant-produced anti-CTLA-4 antibody 2C8 mAb was tested to confirm their binding activities and cross-reactivity to huCTLA-4/His **(A)** or mCTLA-4/His **(B)**. The plant-produced anti-PD-1 and human IgG1 were used in parallel assays as negative controls. The data are presented as mean ± SD of three independent experiments.

The affinity of plant-produced anti-CTLA-4 2C8 mAb to purified CTLA-4 recombinant proteins was investigated by ELISA. Plates were coated either with human CTLA-4 or mouse CTLA-4 and then treated with increasing concentrations of plant-produced anti-CTLA-4 antibody, alongside with plant-produced anti-PD-1 (Rattanapisit et al., 2019) and human IgG1 isotype antibody as negative controls. Specific and dose-dependent binding to CTLA-4 proteins was observed for our plant-produced anti-CTLA-4 2C8 antibody (**Figures 4A, B**), whereas plant-produced anti-PD-1 antibody and human IgG1 did not elicit any binding as expected. Moreover, plant-produced 2C8 mAb was able to recognize both human and mouse CTLA-4 his-tagged proteins. Altogether, the anti-CTLA-4 2C8 mAb produced in *N. benthamiana* is deemed to be functional as it exhibits effective binding and human-mouse cross-reactivity to its target.

**Figure 5:**
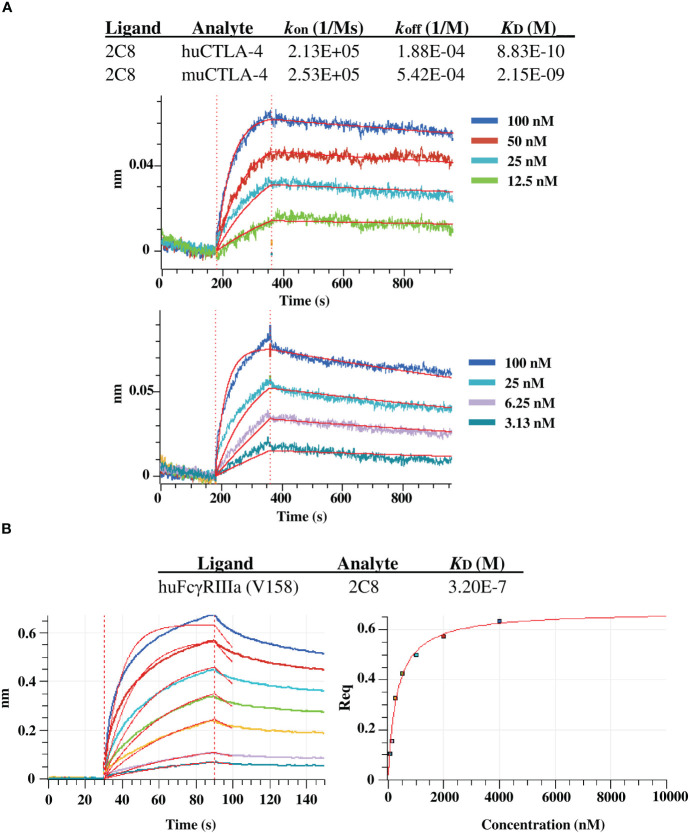
BLI binding analysis of plant-produced anti-CTLA-4 2C8 mAb. **(A)** BLI kinetic analysis of anti-CTLA-4 2C8 against huCTLA-4 (top) and muCTLA-4 (bottom). **(B)** BLI steady state analysis of anti-CTLA-4 2C8 against huFcγRIIIa (V158).

We further evaluated the binding kinetics using BLI with huCTLA-4 and muCTLA-4. The plant-produced 2C8 mAb demonstrated high binding affinity with human CTLA-4 and mouse CTLA-4 targets, having a subnanomolar to low nanomolar equilibrium dissociation constant (*K*_D_) of 8.8×10^-10^ M and 2.2×10^-9^ M (**Figure 5A**). In addition, we also characterized the plant-produced 2C8 IgG1 binding to one of the Fcγ receptors using BLI and obtained *K*_D_ values from steady-state analysis. Data showed that the plant-produced IgG1 exhibited binding affinity for FcγRIIIa (V158) with a *K*_D_ of 3.2×10^-7^ M (**Figures 5B**). Collectively, these results confirmed the high binding affinity of plant-produced 2C8 for CTLA-4 protein and its ability to bind FcγR.

### Plant-produced anti-CTLA-4 2C8 mAb exhibits antitumor activity *in vivo*

**Figure 6:**
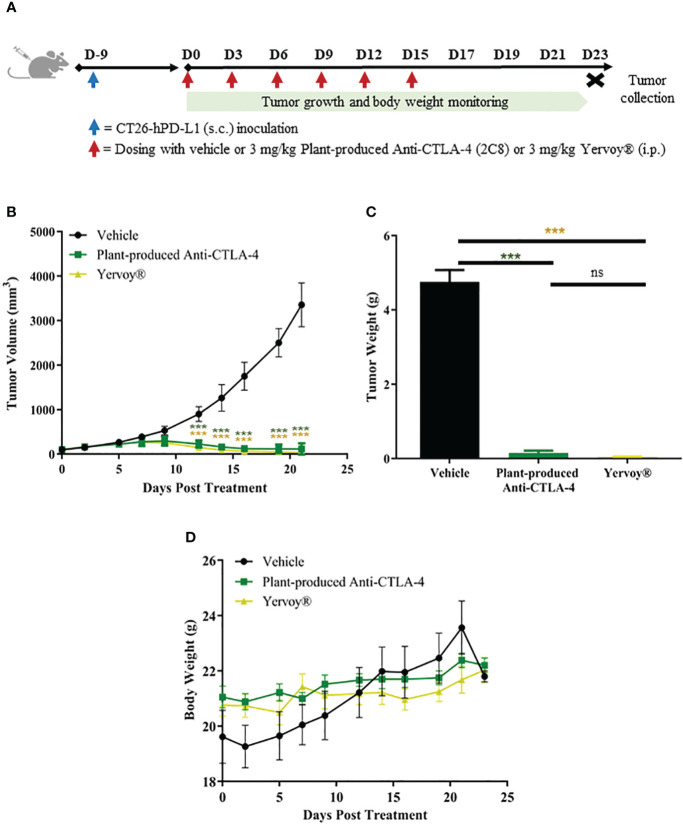
*In vivo* antitumor activity of plant-produced anti-CTLA-4 2C8 mAb. **(A)** Schematic representation of the experimental design used to evaluate antitumor effects in mice model. In this experiment, syngeneic humanized mice bearing CT26-hPD-L1 colon cancer were treated with plant-produced anti-CTLA-4 (3 mg/kg) or commercial anti-CTLA-4 (Yervoy^®^) (3 mg/kg) or vehicle. The generated graphs show **(B)** tumor volume changes over time, **(C)** tumor weight data of mice in different treatment groups, and **(D)** body weight changes during evaluation period. The data are presented as mean ± SD. ns: not significant and ***p < 0.001 by one way ANOVA test, the *post-hoc* test was LSD.

The antitumor efficacy of plant-produced anti-CTLA-4 2C8 mAb was evaluated in BALB/c-hPD-1/hPD-L1/hCTLA-4 mice. The humanized mice were subcutaneously implanted with mouse colon CT26-hPDL-1 tumor cells. The plant-produced anti-CTLA-4 or commercial anti-CTLA-4 (Yervoy^®^) or 1X PBS (vehicle) was administered following the dosage regimen shown on **Figure 6A**. Tumor growth inhibition based on tumor volume (TGI_TV_) and tumor weight (TGI_TW_) were monitored to evaluate the anti-tumor effect. As shown in **Figure 6B**; **Supplementary Table 2**, treatment with plant-produced anti-CTLA-4 at 3 mg/kg significantly regressed the tumor volume (TGI_TV_ = 96.58%) relative to the vehicle group (*p* < 0.001). Furthermore, our plant-produced anti-CTLA-4 antibody demonstrated comparable tumor volume reduction to those observed in the Yervoy^®^ group at same dose (TGI_TV_ = 98.83%). At the conclusion of the study, tumors were collected and weighed. Data was used as a reference for the evaluation of antitumor effect. As shown in **Figure 6C** and summarized in **Supplementary Table 3**, results revealed that treatment with plant-produced anti-CTLA-4 significantly repressed tumor weight (TGI_TW_ = 96.93%) similarly to that of Yervoy^®^-treated group (TGI_TW_ = 99.20%). Hence, our findings demonstrated that the plant-produced anti-CTLA-4 2C8 mAb elicited strong antitumor responses and established similar degree of tumor growth inhibition with the commercially available anti-CTLA-4 drug. Meanwhile, tumor-bearing mice also showed good safety and tolerability to continuous administration of plant-produced anti-CTLA-4 antibody since no apparent body weight loss (>10%) were observed throughout the experiment (**Figure 6D**).

## Discussion

The evasive mechanisms and immunosuppressive microenvironment of most malignant cells provided monumental strides for cancer immunotherapy. Several immunotherapeutic maneuvers have been developed, with effective ICIs receiving most attention. This treatment aims to redirect the host immune system to eliminate cancer via., blockade of immunological checkpoints. Seven first-generation ICIs have been marketed and entered clinical use as of this writing, while over 5,000 trials currently being conducted (Upadhaya et al., 2022). These ICIs have demonstrated remarkable clinical efficacy in an array of recalcitrant carcinomas, including aggressive melanoma, difficult-to-treat lymphomas, and some cancers of the lung, liver, and kidney. Although minority of cancer patients benefitted from such treatments (Taefehshokr et al., 2022), ICIs have pushed the frontiers in cancer care, joining the ranks of conventional therapies. According to a pooled-analysis of anti-CTLA-4 Ipilimumab, more than 20% of treated patients with unresectable or metastatic melanoma experienced long-term survival for up to 10 years and suggested no cancer recurrence thereafter (Schadendorf et al., 2015). Nevertheless, despite favorable outcomes, the expensive cost of ICIs constraints access to and affordability of treatment.

The absolute cost of cancer medications is undoubtedly a crucial and growing global problem. While cancer occurs in all populations, the low- and middle-income countries suffer a double burden due to limited access to treatment. As a result, numerous initiatives to overcome these barriers must be prioritized. The advent of recombinant technology has transformed the standard of care regimens for cancer and a wide range of diseases. Currently, majority of the approved recombinant biopharmaceuticals, including the commercially available ICIs, are routinely produced in mammalian cell cultures. Mammalian cells remain to be the preferred platform; however, this system is rather expensive and requires complex culture conditions (Schillberg and Finnern, 2021). Plants seem to be a desirable alternative and inexpensive method for producing highly valuable proteins. It is well known that economic benefits, speed, and simplicity of scale-up drove concerted attention towards plant systems. The emergence of COVID-19 pandemic intensified the applications of plant platforms with the development of effective vaccine candidates and mAbs against the rapidly spreading SARS-CoV-2 and its variants (Shanmugaraj et al., 2020b; Khorattanakulchai et al., 2022). To this day, many plant-made mAbs are in various stages of development and hold promises for treating a plethora of infections as well as cancer. For example, Ma et al. (Ma et al., 2015) successfully produced P2G12 mAb from transgenic tobacco plants and tested it in a phase I clinical trial as an anti-HIV microbicidal agent. Safety evaluation of plant-based P2G12 in 11 volunteers revealed that no side effects or adverse immune effects were observed following vaginal administration. Furthermore, a tobacco-derived mAb against the *Bacillus anthracis* protective antigen demonstrated toxin-neutralizing activity *in vitro* and *in vivo* (Hull et al., 2005). More importantly, a non-glycosylated version of this mAb was found to be more effective in protecting mice and primates contracted with *B. anthracis* (Mett et al., 2011). In the case of cancer, manufacturing of Rituximab and Trastuzumab in *N. bethamiana* resulted in the development of biosimilar plant products that were as effective as the original drug in terms of inducing ADCC, inhibiting cancer cell proliferation and reducing tumor growth (Komarova et al., 2011; Holtz et al., 2015).

CTLA-4 is regarded as one of the best characterized inhibitory immune checkpoints. Engagement of CTLA-4 receptor to CD80 and CD86 ligands mediate negative co-stimulatory signal, impeding T cell effector functions and abrogating antitumor immune responses. It is widely known that anti-CTLA-4 antibodies block this checkpoint pathway by specifically binding to CTLA-4, disrupting ligand-receptor interactions and then T cell activation. Ipilimumab is the only approved antibody-based therapy produced in CHO cells with some limitations, such as production costs, sterility requirements, and contamination risks (Siddiqui and Rajkumar, 2012; Dhara et al., 2018). To facilitate the economical production of ICI, we produced an anti-CTLA-4 antibody in *N. benthamiana* plants for effective cancer immunotherapy. Here, 2C8 heavy chain and light chain genes were inserted into a plant expression vector and coordinately co-expressed in tobacco leaves. The reliability of *N. benthamiana* to produce functional ICIs has been proven in our earlier reports (Rattanapisit et al., 2019; Phakham et al., 2021; Phetphoung et al., 2022). Similarly, we have adopted an *Agrobacterium*-mediated expression for the production of anti-CTLA-4 checkpoint inhibitor. Based on the results, the infiltrated leaves at 2 dpi showed the least damage while apparent necrosis and wilting were observed at 3-6 dpi. In line with previous studies (Boonyayothin et al., 2022), leaf damage and/or necrosis was observed when a recombinant antibody was transiently expressed, whereas an *Agrobacterium* culture containing no antibody showed no response after infiltration. Relevant literatures also showed similar responses for the expression of recombinant proteins, for example hemagglutinin (Matsuda et al., 2012), hepatitis B (Huang et al., 2008), human growth hormone (Gils et al., 2005), and tumor associated MUC1 peptide (Pinkhasov et al., 2011). The expression level of anti-CTLA-4 antibody increased until 4 dpi, accumulating up to 39.65 ± 8.42 µg/g fresh weight, and then decreased on later dpi. This demonstrates influence of transfection time and necrosis with protein yield (Hamorsky et al., 2015) and suggests that protein expression may be less optimal from 5 dpi and later. Western blotting was also carried out to verify antibody expression in *N. benthamiana* crude extracts. The anti-CTLA-4 2C8 mAb was successfully expressed as expected and was only detected in infiltrated plant crude extracts. According to the obtained results, the anti-CTLA-4 antibody was optimally produced in *N. benthamiana* at 4 dpi, which is an undoubted advantage over transgenic expression (Ma et al., 2015) and mammalian expression (Ahmadi et al., 2017). For antibody purification, protein A-based affinity chromatography was used. SDS-PAGE and western blot analyses revealed that the purified plant-produced anti-CTLA-4 2C8 mAb assembled into its tetrameric isoform under a non-reduced condition whereas heavy chain and light chain monomers were observed in the presence of a reducing agent. Plant systems have distinctive *N*-glycosylation machinery from humans (Strasser et al., 2008) that differ substantially during the late stages of *N*-linked glycan processing. Plants often have high-mannose-type *N*-glycans with Man_5-9_GlcNAc_2_ structure (Rayon et al., 1998). Here, the *N*-glycan profile of plant-produced anti-CTLA-4 depicted high-mannose oligosaccharides, as a result of targeted accumulation in the ER via., SEKDEL peptide sequence. The presence or absence of localization signals causes differences in the *N*-glycosylation of proteins when synthesized in plants (Triguero et al., 2011), with complex-type glycans of β(1,2)-xylose and α(1,3)-fucose residues abundant in plantibodies lacking KDEL and oligomannose-type glycans predominant in plantibodies with KDEL, as seen in our study and others (Ko et al., 2003; Triguero et al., 2005). These oligomannosidic glycans found in SEKDEL-containing plant glycoproteins are non-immunogenic as reported elsewhere (Sriraman et al., 2004). Additionally, in previously published *N*-glycan analyses of anti-PD-1/PD-L1 mAbs by our team (Phakham et al., 2021; Phetphoung et al., 2022), *N-*glycosylation patterns differed among antibodies produced in plants to that of mammalian cells, at which authentic plant glycan structures were derived in plant-produced antibodies but absent in aglycosylated Atezolizumab (Tecentriq^®^) while only mammalian-type glycans were observed in commercial Pembrolizumab (Keytruda^®^). Meanwhile, glycoengineered plants offers another approach to diminish or abolish plant-specific *N*-glycan repertoire (Fischer et al., 2021).

The ability of purified plant-produced 2C8 mAb to bind to its target antigen has been demonstrated *in vitro.* Using human and mouse CTLA-4 proteins, the plant-produced anti-CTLA-4 antibody exhibited concentration-dependent binding. In contrary, both controls such as non-related anti-PD-1 antibody and human IgG1 isotype only showed background binding. Likewise, BLI analysis confirmed subnanomolar or low nanomolar affinity for human and mouse CTLA-4 proteins, demonstrating the successful production of a functional mAb in plants with binding activity and mouse-human cross-reactivity. The binding properties of our plant-produced 2C8 differ from that of Ipilimumab, owing to its low cross-reactivity for mouse CTLA-4 (He et al., 2017; Li et al., 2020; Passariello et al., 2020), which we have not confirmed. Moreover, the Fcγ receptor engagement of plant-produced 2C8 was also investigated by BLI. The anti-CTLA-4 antibody exhibited sub-micromolar binding for FcγRIIIa (V158) typical for IgG1 and is consistent with previous studies (Gombos et al., 2018), suggesting potential effector function. Moreover, the presence of plant mannosidic *N*-glycans did not significantly alter the binding affinity of plant-produced mAb to its CTLA-4 target, which is in line with other earlier findings (Phakham et al., 2021; Phetphoung et al., 2022), and also to one of the FcγRs. Previous reports have indicated the relevance of FcγR-dependent functions via., ADCC and ADCP in anti-CTLA-4 therapies (Arce Vargas et al., 2018; Ingram et al., 2018), which contributes to antitumor responses in mouse tumor models. However, preclinical design of an anti-CTLA-4 IgG2 isotype to minimize Fc effector function (Hanson et al., 2004) implicates the unclear roles of FcγRs in the activity of mAbs.

In order to elucidate a preclinical rationale for plant-based checkpoint inhibitors, we preliminarily tested the antitumor activity of our plant-produced anti-CTLA-4 antibody *in vivo*. Here, we utilized a PD-1/PD-L1/CTLA-4 humanized BALB/c mice with established CT26-hPD-L1 colon carcinoma. The CT26 colon cancer cell line is categorized to be highly immunogenic (Lechner et al., 2013) and has been used in a variety of *in vivo* studies, including our current study. Based on the results, we found that plant-produced anti-CTLA-4 2C8 elicited significant antitumor efficacy in syngeneic CT26-hPD-L1 tumor model. More importantly, treatment with plant-produced anti-CTLA-4 reduced the tumor size and repressed the tumor weight comparably with the mammalian cell produced anti-CTLA-4 (Yervoy^®^). In contrast, treatment with vehicle failed to control tumor growth. These data reveal the direct effects of anti-CTLA-4 antibodies in the reversal of CTLA-4-induced T cell tolerance and anergy (Peggs et al., 2009; Pardoll, 2012). In particular, our plant-produced anti-CTLA-4, together with Yervoy^®^, blocked the CTLA-4 function on tumor cells, leading to a marked inhibition of tumor growth as a result of T cell activation. Interestingly, the syngeneic murine tumor model shown substantial sensitivity to anti-CTLA-4 immunotherapy, wherein our plant-produced anti-CTLA-4 was as sensitive as the commercial anti-CTLA-4 in suppressing growth of tumors positive for PD-L1 expression. In line with our study, the efficacy of anti-CTLA-4 antibody was reported in other murine tumor models of colon cancer (*ie.*, CT26, Colon 26, and MC38) which showed high sensitivity and durable responses to CTLA-4 blockade (Grosso and Jure-Kunkel, 2013; Wei et al., 2017; Sato et al., 2021). On the other hand, treatment-related toxicity was assessed based on body weight. An obvious decrease in the body weight has been associated with the prospective occurrence of toxicity and side effects in treated mice, which eventually undermines their chance of survival (Chapman et al., 2013). In the present study, no significant body weight changes were recorded in any of the treatment groups such that serious body weight loss of over 10% was not evident following the treatment of either anti-CTLA-4 antibodies or vehicle. These results, in turn, translate into the favorable safety and tolerability of the plant-produced anti-CTLA-4 treatment. Although notable differences in body weight may not completely establish overall treatment-associated toxicities, preliminary results reported here provide sufficient basis for future exploration in non-clinical toxicology studies.

Altogether, a recombinant anti-CTLA-4 2C8 antibody has been successfully produced in *N. benthamiana*. This plant-derived antibody cross-reacts to both human and mouse CTLA-4, binds to FcγRIIIa (V158) and elicits excellent antitumor effects in syngeneic mouse model. To our knowledge, this study addresses the first preclinical assessment of a plant-produced anti-CTLA-4 immunotherapy. More so, the cost-effective production of plant-based immune checkpoint inhibitor further affirms the suitability of the current platform to relieve financial strain and improve treatment accessibility in the developing countries.

## Data availability statement

The original contributions presented in the study are included in the article/**Supplementary Material**. Further inquiries can be directed to the corresponding author.

## Ethics statement

The animal study was approved by the Institutional Animal Care and Use Committee (IACUC) of GemPharmatech Co., Ltd., China. The study was conducted in accordance with the local legislation and institutional requirements.

## Author contributions

WP and CJIB conceived the study. NK and KR performed gene synthesis and cloning. CJIB performed antibody expression, purification, quantification and binding assays. CJIB and NP performed size exclusion chromatography. RS performed N-glycan analysis. ST and PS-S performed BLI analysis. HS performed *in vivo* antitumor assays. All authors contributed to the article and approved the submitted version.
