# Supplementary material for: Antitumor effect of plant-produced anti-CTLA-4 monoclonal antibody in a murine model of colon cancer
Source: Front Plant Sci. 2023 Aug 29;14:1149455. doi: 10.3389/fpls.2023.1149455 (PMC10497774; doi:10.3389/fpls.2023.1149455)
Supplement: Supplementary file 1 [file Table_1.docx]

Supplementary Material

**Antitumor effect of plant-produced anti-CTLA-4 monoclonal antibody in a murine model of colon cancer**

**Christine Joy I. Bulaon^1,2,3^, Narach Khorattanakulchai^4^, Kaewta Rattanapisit^4^, Hongyan Sun^5^,** **Nuttapat Pisuttinusart^1,2,3^, Richard Strasser ^6^, Shiho Tanaka^7^, Patrick Soon-Shiong^7^ and Waranyoo Phoolcharoen^1,2*^**

*** Correspondence:** Waranyoo Phoolcharoen: Waranyoo.P@chula.ac.th

# Supplementary Tables

# Supplementary Table 1: The expression levels of 2C8 antibody in *N. benthamiana* on 2, 3, 4, 5, and 6 days after agroinfiltration

| Day of harvest | Pembrolizumab expression (µg/g FW) | | | Average  (µg/g FW) |
| --- | --- | --- | --- | --- |
|  | #1 | #2 | #3 |  |
| D2 | 22.00 | 25.14 | 30.84 | 26.00 ± 4.48 |
| D3 | 22.68 | 40.08 | 39.03 | 33.93 ± 9.76 |
| D4 | 36.35 | 49.22 | 33.39 | 39.65 ± 8.42 |
| D5 | 18.56 | 24.49 | 27.28 | 23.44 ± 4.45 |
| D6 | 16.14 | 17.65 | 11.62 | 15.14 ± 3.14 |

2C8 expression levels were measured using ELISA and reported as µg/g leaf fresh weight (FW). Three leaf samples (#1, #2, #3) were harvested on days 2, 3, 4, 5, and 6 post-infiltration (D2, D4, D6, D8) and pooled for analysis (reported as mean ± SD).

**Supplementary Table 2:** Statistical analysis of tumor volume changes in different groups

| **Group** | **Tumor volume (mm^3^)** | **TGI_TV_ (%)** | ***p* value**  **(vs. Vehicle)** | ***p* value**  **(vs. Yervoy®)** |
| --- | --- | --- | --- | --- |
| Vehicle | 3352.97 ± 201.59 | - | - | - |
| Plant-produced Anti-CTLA-4 | 116.98 ± 51.8 | 96.58% | <0.001 | 0.806 |
| Yervoy® | 38.12 ± 8.96 | 98.83% | <0.001 | - |

Note: Data were shown as mean ± SD; Statistics performed using one-way ANOVA test, the post-hoc test was LSD, ***: *p*<0.001.

# Supplementary Table 3: Statistical analysis of tumor weight in different groups

| **Group** | **Tumor weight (g)** | **TGI_TW_ (%)** | ***p* value**  **(vs. Vehicle)** | ***p* value**  **(vs. Yervoy®)** |
| --- | --- | --- | --- | --- |
| Vehicle | 4.7548 ± 0.3183 | - | - | - |
| Plant-produced Anti-CTLA-4 | 0.1462 ± 0.0651 | 96.93% | <0.001 | 0.814 |
| Yervoy® | 0.0380 ± 0.0096 | 99.20% | <0.001 | - |

Note: Data were shown as mean ± SD; Statistics performed using one-way ANOVA test, the post-hoc test was LSD, ***: *p*<0.001.
